# Genetic Loss of VGLUT1 Alters Histogenesis of Retinal Glutamatergic Cells and Reveals Dynamic Expression of VGLUT2 in Cones

**DOI:** 10.3390/brainsci15091024

**Published:** 2025-09-22

**Authors:** Sriparna Majumdar, Vincent Wu

**Affiliations:** 1 Department of Biology, Stanford University School of Medicine, Stanford, CA 94305, USA; 2 Computer Science Department, City College of San Francisco, San Francisco, CA 94112, USA; 3 Kaiser Permanente Redwood City Medical Center, Redwood City, CA 94063, USA

**Keywords:** mouse, retina, development, extrinsic, glutamate, VGLUT1, VGLUT2, VGLUT3, melanopsin, bipolar, amacrine, ganglion

## Abstract

**Background/Objectives**: Glutamatergic neurotransmission is essential for the normal functioning of the retina. Photoreceptor to bipolar and bipolar to ganglion cell signaling is mediated by L-glutamate, which is stored in and released from vesicular glutamate transporter 1 (VGLUT1) containing synaptic vesicles. VGLUT1 is expressed postnatally, P2 onwards, and is required for the glutamatergic retinal wave observed between P10 and P12 in the developing mouse retina. P9–P13 postnatal age is critical for retinal development as VGLUT1 expressing ribbon synapses activate in the outer and inner plexiform layers, and rod/cone mediated visual signaling commences in that period. Although it has been hypothesized that glutamatergic extrinsic signaling drives cell cycle exit and initiates cellular differentiation in the developing retina, it is not clear whether intracellular, synaptic, or extrasynaptic vesicular glutamate release contributes to this process. Recent studies have attempted to decipher VGLUT’s role in retinal development. Here, we investigate the potential effect of genetic loss of VGLUT1 on early postnatal histogenesis and development of retinal neural circuitry. **Methods**: We employed immunohistochemistry and electrophysiology to ascertain the density of glutamatergic, cholinergic, and dopaminergic cells, spontaneous retinal activity, and light responses in VGLUT1 null retina, and contrasted them with wildtype (WT) and melanopsin null retina. **Results**: We have demonstrated here that VGLUT1 null retina shows signs of age dependent retinal degeneration, similar to other transgenic mice models with dysfunctional photoreceptor to bipolar cell synapses. The loss of VGLUT1 specifically alters glutamatergic cell density and morphological maturation of retinal ganglion cells. Moreover, VGLUT2 expression is lost in the majority of VGLUT2 cones in the absence of VGLUT1 coexpression, except when VGLUT2 coexpresses transiently with VGLUT3 in these cones, or when VGLUT1 null mice are dark reared. **Conclusions**: We present the first evidence that synaptic or extrasynaptic postnatal glutamate release from VGLUT1 containing vesicles impacts histogenesis of glutamatergic cells, pruning of retinal ganglion cell dendrites and VGLUT2 expression in cones.

## 1. Introduction

L-glutamate is the most important excitatory neurotransmitter in the central nervous system. More than half of the synapses in the brain are glutamatergic [1]. The retinal photoreceptors rods and cones use glutamate to transmit the visual input to the postsynaptic bipolar and horizontal cells in the outer plexiform layer (OPL); bipolar cells in turn use glutamate to relay the signal to ganglion and amacrine cells in the inner plexiform layer (IPL) [2]. Glutamatergic signaling via retinal ganglion cell projections underlies the transmission of preprocessed retinal information to the lateral geniculate nucleus (LGN) of the thalamus and accessory visual systems, notably optic pretectum, superior colliculus, and suprachiasmatic nucleus of the hypothalamus [3,4]. Vesicular glutamate transporters (VGLUTs) are required to fill the synaptic vesicles with glutamate. VGLUT isoforms VGLUT1-3 (genes *Slc17a6-8*) are expressed in distinct cell populations in the mammalian retina and in the brain [5,6]. Photoreceptors and bipolar cells express VGLUT1; ganglion cells and a small subset of cones express VGLUT2, and a subtype of glycinergic amacrine cells expresses VGLUT3 [5,7,8]. A similar complementary expression pattern of VGLUTs is also seen in the central nervous system. The neocortex, cerebellar cortex, and hippocampus predominantly express VGLUT1, whereas the thalamus and brainstem almost exclusively express VGLUT2 [9,10]. VGLUT3 is expressed in select cell types coexpressing another excitatory or inhibitory neurotransmitter transporter, and is the main VGLUT in the auditory pathway. VGLUT1 null mice are impaired in their scotopic and photopic vision, VGLUT3 null mice are deaf, VGLUT2 null mice die at birth, and VGLUT2 deficient mice are unable to acquire neuropathic pain [10,11,12]. All the VGLUT null or conditional knockout mice are susceptible to chronic seizure. These findings signify the relative indispensability of all the VGLUT types in sensory processing.

Developmental appearance of the three VGLUTs follows a unique timeline. VGLUT2 is expressed in the central nervous system of mice prenatally, starting embryonic day 9.5 (E9.5), and is the only VGLUT in the retina until P2, when VGLUT1 starts to express in the photoreceptor presynaptic terminals, followed by its expression in the bipolar cell presynaptic terminals starting at P5 [1,5,13]. VGLUT1 is expressed nowhere prenatally except for the olfactory cortex [14]. VGLUT3 starts to express at E18 in the nervous system and at P7 in the mouse retina [8,14]. A minority of cells in the nervous system coexpress more than one VGLUT, either in separate synaptic vesicles, or in the same vesicles. About 10% of all cones coexpress VGLUT1 and VGLUT2 [7]. Sparse coexpression of VGLUT1 and VGLUT2 is evidenced throughout the brain, including in the spinal cord, cerebellar mossy fibers, hippocampus, and calyx of held [9,14,15]. The pattern of expression of three VGLUTs changes over the course of development. VGLUT2 transiently expresses in striatum and parallel fibers in the cerebellum and switches to VGLUT1 postnatally [9,14]. VGLUT3 expresses transiently in layer 6 of cortex at P7, whereas it is strongly expressed in layer 2 of adult mouse cortex [14]. Such transient expression of VGLUTs was not reported earlier in the mammalian retina.

During early prenatal development, neurotransmitters and small molecule messengers, secreted in the extracellular spaces, drive the developmental cascades through interacting with their cognate receptors expressed on the progenitor cell membranes. These diffusible extrinsic signals change to initiate cell cycle exit, which is almost always accompanied by a switch to a cell intrinsic signaling mechanism required for cellular differentiation [16]. Intrinsic signaling molecules like micro RNAs and transcription factors selectively turn on cell fate specific genes. Glutamate serves as one of the extrinsic signals in the developing nervous system. The mechanism of glutamate release in the developing retina and brain is still not clear, albeit the prenatal expression of VGLUT2 everywhere in the central nervous system. The excitatory amino acid transporter (EAAT) family of transmembrane glutamate transporters expresses in retinal progenitors prenatally. They may actively transport glutamate out of the cell, thereby establishing the glutamate mediated extrinsic signal [1,17]. We have shown that VGLUT2 mediated glutamatergic neurotransmission by melanopsin containing intrinsically photosensitive retinal ganglion cells (ipRGCs) is required for neonatal photoaversion behavior especially at P8 or P9, before VGLUT1 mediated rod/cone pathway activates [18]. ipRGCs are born prenatally and are functionally active beyond E15 [19]. It remains to be tested whether VGLUT2 expressed in ipRGCs and other retinal ganglion cells contributes to prenatal extrinsic glutamatergic signaling.

Retinal ganglion cells periodically activate in the developing pre and postnatal retina, and participate in the correlated burst of action potential firing, known as retinal waves. Stage I retinal waves are seen between E17 and P1 and are mediated by gap junctional communication. Stage II waves are cholinergic. They dominate retinal waves between P2 and P9, followed by the stage III glutamatergic wave at P10–P12 that guides rod/cone to bipolar and bipolar to ganglion cell ribbon synapse formation [1]. VGLUT1 is critically needed for the stage III wave. VGLUT1 null mice are impaired in the stage III glutamatergic wave and rod/cone mediated image forming vision [12,20]. Biswas et al. have recently shown that postnatal retinal angiogenesis and blood retinal barrier maturation require VGLUT1 mediated glutamatergic neurotransmission [21]. These findings together emphasize VGLUT1’s role in postnatal retinal development. We show here that the loss of postnatal VGLUT1 mediated glutamate release in the retina causes age related photoreceptor degeneration, and permanently alters bipolar, VGLUT3 amacrine, and retinal ganglion cell density. Moreover, VGLUT1 mediated glutamate release promotes the maturation of dendritic arbors of retinal ganglion cells, as the genetic loss of VGLUT1 causes incomplete pruning of retinal ganglion cell dendrites. The majority of VGLUT2 positive cones fail to express VGLUT2 in the absence of VGLUT1. Transient VGLUT3 co expression or dark rearing restores VGLUT2 expression in the VGLUT2 cones of VGLUT1 null retina.

## 2. Materials and Methods

### 2.1. Animals

Germline *Vglut1* knockout (*Vglut^−/−^*, VGLUT1 null, or KO) mice were generated as reported previously [12]. Transgenic mice expressing EGFP in ON bipolar cells (*L7-egfp*) were obtained from M. Yuzaki [22]. Three VGLUT3 Het and null (*Vglut3^+/−^* and *Vglut3^−/−^*) mice were a gift from the Robert Edwards Lab [11]. C57Bl/6, *Thy1-yfp*, melanopsin null (*Opn4^−/−^*), and melanopsin-GFP (*Opn4^GFP^*) mice were procured by Copenhagen Lab ([19], Jackson Laboratories, Sacramento, CA, USA). *VGlut1^+/−^*; *Thy1-yfp^+/−^*, *VGlut1^−/−^*; *Thy1-yfp^+/−^*, *VGlut1^+/−^*; *L7-egfp^+/−^*, and *VGlut1^−/−^*; *L7-egfp^+/−^* mice strains were generated in our animal house by crossing *VGlut1^+/−^* with *Thy1-yfp^+/−^* and *L7-egfp^+/−^* strains. Animals were fed ad libitum and housed under a 12 h light/dark schedule. All animal procedures were approved by the University of California, San Francisco Institutional Animal Care and Use Committee, and conformed to National Institutes of Health Guide for the Care and Use of Laboratory Animals and the Society for Neuroscience Policy on the Use of Animals in Neuroscience Research (grant NIH EY01869, Tenure 1 February, 30 June 1977, 2015). The VGLUT1 null (*Vglut1^−/−^*, KO) retina were compared to VGLUT1 Heterozygous (*Vglut1^+/−^*, Het) or wildtype (*Vglut1^+/+^*, WT) littermate retina, unless otherwise mentioned. When unattended, VGLUT1 null mice died by postnatal day 21. Milk based extra enriched food helped them thrive until about 1 year. The mice were blind and would sometimes have seizures. They were housed together with one Het/WT littermate. Often, they suddenly fell very sick and required to be euthanized. That is one reason why we could not study the expression of all proteins of interests at the exact same age. The data presented here are broadly grouped into 4 age categories, based on the published literature on the timeline of retinal development, experimental aim, and tissue availability: early postnatal developing age (P8–P14, until eye opening), juvenile developing age (P15–P30), developed young adults (1–4 months), and adult/aged (4–11 months). The exact age in these 4 broad categories somewhat varied from experiment to experiment and is presented as is in the images, statistics, and plots. Both male and female data were combined for each genotype. Data from *Vglut1^+/+^* and *Vglut1^+/−^* were comparable and hence were grouped together as VGLUT1 WT.

### 2.2. Tissue Preparation and Immunohistochemistry

Briefly, the mice were sacrificed after isoflurane anesthesia followed by cervical dislocation, and their eyes were harvested. Their corneas were removed and eyes were immediately fixed in 4% (*v*/*v*) paraformaldehyde (PFA) in 0.1 M PBS for 15–30 min at room temperature. The eyes were then rinsed in 0.1 M PBS, and cryoprotected by incubating in 10%, 20%, and 30% sucrose in 0.1 M PBS solutions at room temperature for 1 h each, embedded in ornithine carbamyl transferase (OCT; Tissue-Tek, Elkhart, IN, USA), and frozen on dry ice. Vertical sections of the retina were cut with a cryostat at 20–30 μm thickness and stored frozen at −20 °C.

For immunohistochemistry, retinal sections were incubated overnight at room temperature in a primary antibody with 0.5% Triton X-100 and 10% bovine serum albumin. In some experiments, antibodies were diluted in a solution containing 0.5% Triton X-100, 15% bovine serum albumin, and 3% normal donkey serum. 1% Triton X-100 was used instead of 0.5% Triton X-100 for immunostaining whole mounted retinas. The primary antibody concentrations and their sources are summarized in Table 1. The retinal sections or whole mounts were washed 3 times, 10 min each in 0.1 M PBS the next day and incubated in secondary antibodies conjugated to AlexaFluor^®^ 488, 594, or 647 (1:500 dilution) for 1–3 h at room temperature. After a 3 times, 10 min each wash in 0.1 M PBS, they were mounted with Vectashield (Vector Laboratories, Burlingame, CA, USA), cover slipped, and stored at 4 °C.

### 2.3. Fluorescence Detection and Image Processing

Confocal images were captured using a Zeiss (Thornwood, NY, USA) Pascal confocal microscope. Image scale was calibrated and brightness and contrast were adjusted. The VGLUT1 null mice are 30–40% smaller in body size than their healthy (WT) littermates, although their eyes are not significantly smaller. The mean area of adult (1–9 months) VGLUT1 WT retina is 25.86 ± 2.93 μm^2^ and null retina is 24.30 ± 2.37 µm^2^. The null retina is 0–15% smaller than the WT retina, with a mean difference of 5.67% (*n* = 4). The cell density values for null retina were reduced by 5.67%, to accommodate this difference. For all statistics, radial sections and whole mounts from 3–7 retinas were used for each age group. Robust morphological differences were observed between WT and VGLUT1 null retina. In most cases, statistical significance was reached with just 3 animals in each group. Unless otherwise mentioned, cell bodies were counted using 20× objective in two 635 μm × 635 μm near-central regions, but in opposite quadrants across from the optic nerve, and averaged. Data are presented as mean ± SEM. Unpaired Student’s *t*-test was used to compare data between different genotypes, and statistical test results are marked with * (*p* < 0.05) or ** (*p* < 0.01). An age dependent variation in cell densities was seen in both WT and VGLUT1 null groups. Therefore, WT and VGLUT1 null data were compared within each age group, and not across age groups. While imaging WT and VGLUT1 null retinas with differential cell densities and protein expression, image acquisition settings were created for the sub-saturating brightness of the brightest region of interests and used for imaging all retinas used for the quantitation of cell densities and fluorescent intensities. Accordingly, retinas with lower cell density or protein expression looked significantly subdued. The image brightness and contrast were later adjusted offline at values that made visualization of all retinas possible, at the cost of making the brightest regions of interest too bright.

### 2.4. Multielectrode Array Recordings

Multielectrode array (MEA-60, Multi Channel Systems, USA) recordings of light-evoked ganglion cell spiking were acquired and analyzed as described previously [34]. Briefly, the MEA chambers consisted of an array of 60 planar electrodes, each having a diameter of 10 μm. They were arranged in eight rows and spaced 100 μm apart. Voltage signals were bandpass filtered at 0.1 Hz to 3 kHz and sampled at 20 kHz (MC_Rack, version 2.0; Multi Channel Systems, USA). Action potential waveforms from high-pass-filtered data (100 Hz lower cutoff) were detected by threshold crossing and clustered based on their first two principal components. Cluster contours in principal components space were either manually selected or derived from a k-means algorithm (OfflineSorter, version 1.3; Plexon Inc., USA). The algorithm eliminated outlier waveforms at a threshold of 1.3 times the mean distance from the calculated cluster center. Obvious automatic sorting errors were corrected for each cluster manually. Time stamp for each action potential of each sorted unit was used to generate peristimulus time histograms and peristimulus spike rasters. Light stimuli were presented from a monitor (Dell Ultrascan, Dell Infotech Inc., USA P780; 100 Hz vertical refresh) and imaged onto the retinal surface at an approximate intensity of 0.35 W/cm^2^.

## 3. Results

**Figure 1:**
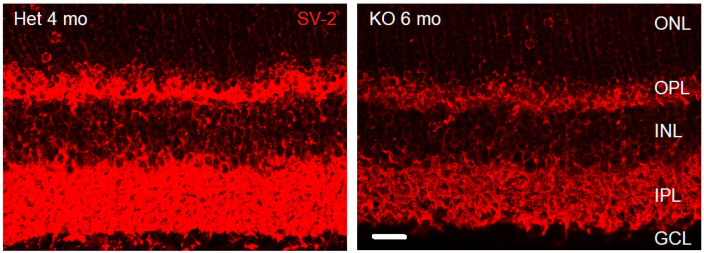
**Synaptic vesicles are reduced in VGLUT1 null retina compared to age matched adult retina.** SV-2 expression in adult VGLUT1 Het and null retina. Scale bar: 10 μm. The brightness and contrast of the images were enhanced for visualizing SV-2 in KO 6 mo retina, which had diminished yet detectable SV-2 expression, presumably at the VGLUT3 mediated glutamatergic, cholinergic, GABAergic, and glycinergic synapses.

This study examines phenotypic and morphological changes in the mice retina triggered by the germline loss of VGLUT1. The first two excitatory synapses involved in the relay of visual signals from photoreceptor outer segments (OS) to the retinal ganglion cells express VGLUT1. Johnson and colleagues showed that adult VGLUT1 null retina had no functional scotopic or photopic b wave, proving that the photoreceptor to bipolar excitatory ribbon synapses were silent in these mice [12]. In the absence of VGLUT1 protein expression and with the loss of glutamatergic signaling at the ribbon synapses, the expression of synaptic vesicle protein SV-2 is reduced in the OPL and IPL of adult VGLUT1 null retina (Figure 1). Consequently, we saw dramatic differences in the retinal morphology due to a reduced vesicular glutamate release in the OPL and IPL. These findings are presented in the following sections. The genetic loss of structural and functional proteins implicated in the formation and maintenance of photoreceptor to bipolar cell synapses induce age related retinal degeneration [35]. Therefore, we first tested whether age related degeneration reminiscent of retinitis pigmentosa was evidenced in VGLUT1 null retina. The results from this first part are outlined in Section 3.1 and Section 3.2. The following Section 3.3 outlines the novel changes in glutamatergic inner retinal cell density in VGLUT1 null retina. Section 3.4 discusses impact of the loss of VGLUT1 mediated glutamate release on retinal ganglion cell dendritic pruning. Section 3.5, Section 3.6 and Section 3.7 highlight dynamic alterations in the expression of VGLUT2 in a subset of cones of VGLUT1 null retina.

### 3.1. VGLUT1 Null Retina Exhibits Loss of Function Induced Progressive Loss of Photoreceptors Similar to Mouse Models of Retinitis Pigmentosa

**Figure 2:**
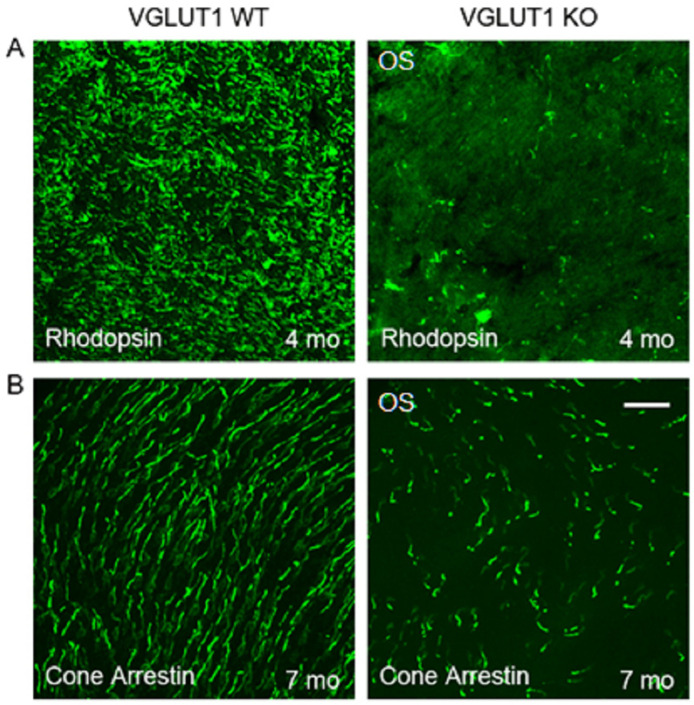
**Photoreceptor outer segments have reduced cell specific marker expression in the adult VGLUT1 null retina.** (**A**) Rhodopsin in the rod outer segments of 4 months old WT and VGLUT1 null (KO) littermate retinas viewed in whole mounts. (**B**) Expression of cone arrestin in 7 months old WT and VGLUT1 null (KO) littermate retinas viewed in whole mounts. Scale bar: 10 μm in (**A**) and 20 μm in (**B**).

Research from other laboratories has established that blinding diseases like macular degeneration, diabetic retinopathy, and retinitis pigmentosa involve dysfunctional photoreceptor to bipolar cell synapses with the progressive loss of photoreceptors and the gradual disorganization of the OPL, leaving the inner retina more or less intact [36]. We noticed similar age related changes in the VGLUT1 null retina. At P30 and older ages, progressively fewer rod and cone outer segments stained for rhodopsin and cone arrestin (not shown). By 4 months age, rhodopsin immunoreactivity is significantly diminished in the central region of the OS of whole mounted VGLUT1 null retina compared to the littermate control (Figure 2A).

**Figure 3:**
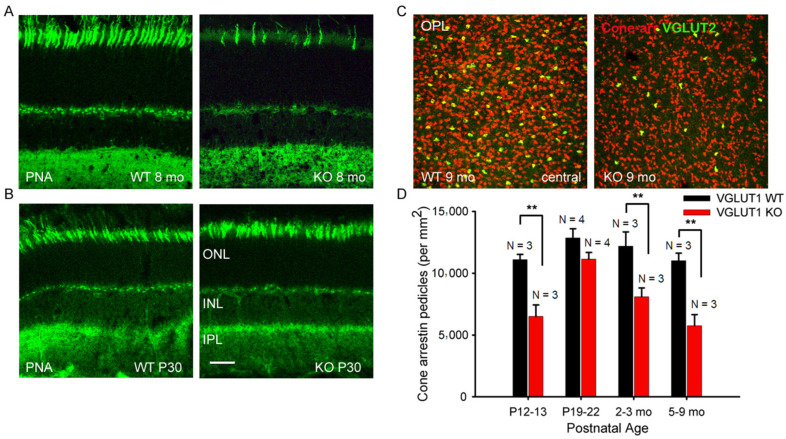
**Gradual, age dependent loss of cone photoreceptor markers in VGLUT1 null retina.** (**A**) Representative images of peanut agglutinin (PNA), a legume lectin known to mark cone outer segments and pedicles, stained in 8 months old WT and VGLUT1 null (KO) littermate retinas. (**B**) PNA staining in the radial sections of P30 WT and VGLUT1 null (KO) littermate retinas. (**C**) Expression of cone arrestin and VGLUT2 in the OPL of whole mounted retinas of 9 months old WT and VGLUT1 null (KO) littermates. (**D**) A comparison of density of cone arrestin positive pedicles in different age groups of WT and VGLUT1 null (KO) mice. Cone arrestin starts to express in a delayed fashion in VGLUT1 null retina. At P12–13, the density of cone arrestin positive pedicles is significantly low (** indicates *p* < 0.01); the density reaches the WT level around P20, beyond which it gradually falls to a significantly lower value, indicating a lack of activity induced gradual loss of cone arrestin protein expression in the VGLUT1 null retina. Scale bar: 20 μm in (**A**,**B**), 50 μm in (**C**). A 318.5 μm × 318.5 μm area or a 112.5 μm × 112.5 μm area was chosen for counting cone arrestin and VGLUT2 pedicles in 2 separate near-central regions in each whole mounted retina and averaged.

A comparable reduction in cone arrestin staining was observed in the OS of whole mounted retina at 7 months or older ages, supporting previous reports that, in the absence of photoreceptor to bipolar cell signaling in the OPL, rods degenerate first, followed by cones (Figure 2B, [36]). Peanut agglutinin (PNA) is a marker of cone photoreceptor outer segments and pedicles. PNA expression is seen in fewer cones in 8 months old VGLUT1 null retina compared to the littermate control (Figure 3A), yet their densities are comparable between WT and KO groups at juvenile developing age of P13–P30, indicating that the PNA positive cone photoreceptors are normally present in the VGLUT1 null retina, with a density comparable to WT retina, but degenerate with age (P30 data are shown in Figure 3B, P13 data are not shown).

**Figure 4:**
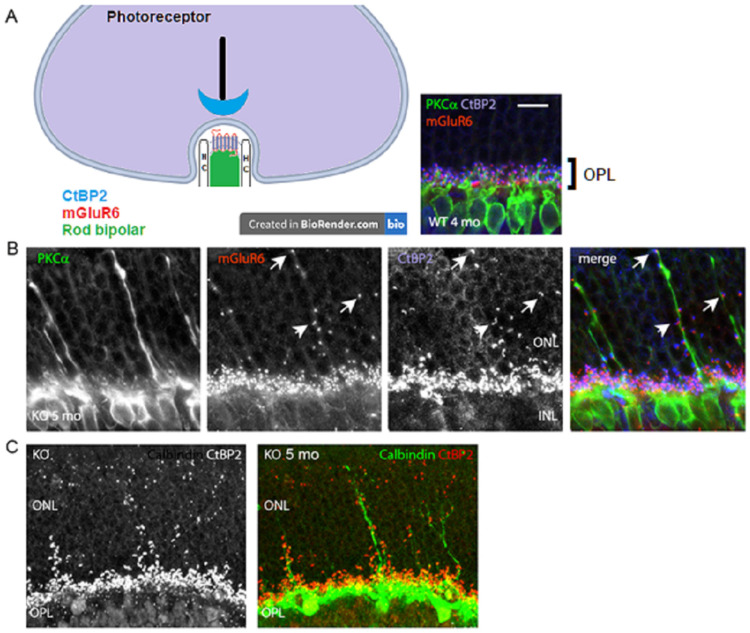
**Ectopic synaptic formations in the ONL of VGLUT1 null retina.** (**A**) Simplified schematic of a rod photoreceptor ribbon synapse, showing the position of presynaptic ribbon associated protein RIBEYE marker CtBP2, postsynaptic rod bipolar cell and horizontal cell (HC) processes, and postsynaptic receptor mGLUR6. (Right inset) CtBP2 puncta are closely associated with mGluR6 puncta at the tip of RBC dendrites in the OPL of 4 months old VGLUT1 WT retina. No CtBP2 or mGLUR6 puncta were observed in the ONL. (**B**) Presynaptic CtBP2 puncta (blue) are associated with postsynaptic mGluR6 (red) puncta at the proliferating RBC dendrite (green) tips and shafts of 5 months old VGLUT1 null retina, suggesting ectopic synaptic assembly. Arrows indicate apposition of CtBP2 and mGLUR6 puncta at three locations on RBC sprouts. (**C**) CtBP2 (red, left) marks ectopic presynaptic sites on the calbindin (green) stained horizontal cell sprouts (right) in 5 months old VGLUT1 null retina. Such calbindin sprouting dendrites are never seen in the age matched WT retinas. Scale bar: 10 μm.

**Figure 5:**
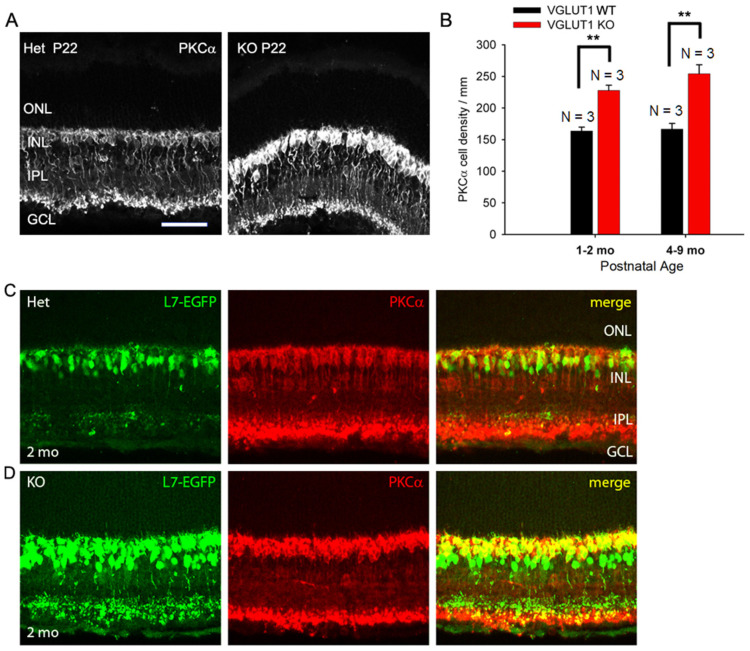
**Density of ON bipolar cells is higher in older VGLUT1 null retina.** (**A**) Rod bipolar cells of P22 WT and VGLUT1 null retinas are stained with the PKCα antibody. (**B**) The cell bodies of rod bipolar cells were counted along a line in the INL in radial sections. There are significantly higher numbers of rod bipolar cells in both juvenile adult and aged VGLUT1 null retina (** indicates *p* < 0.01). (**C**,**D**) GFP and PKCα expression are shown in L7-EGFP expressing WT and VGLUT1 null retinas. There are a significantly higher number of EGFP and PKCα expressing cells in VGLUT1 null retina. No statistics could be calculated for these cells because of their very high density. Scale bar: 50 μm. Incidentally, the levels of PKCα and EGFP protein expression per individual ON cell seemed higher in VGLUT1 null retina. Therefore, VGLUT1 null retinas were imaged first and WT retinas were imaged using VGLUT1 null settings. For presentation purposes, the brightness and contrast of images were adjusted offline after quantitation, making VGLUT1 null images overexposed.

**Figure 6:**
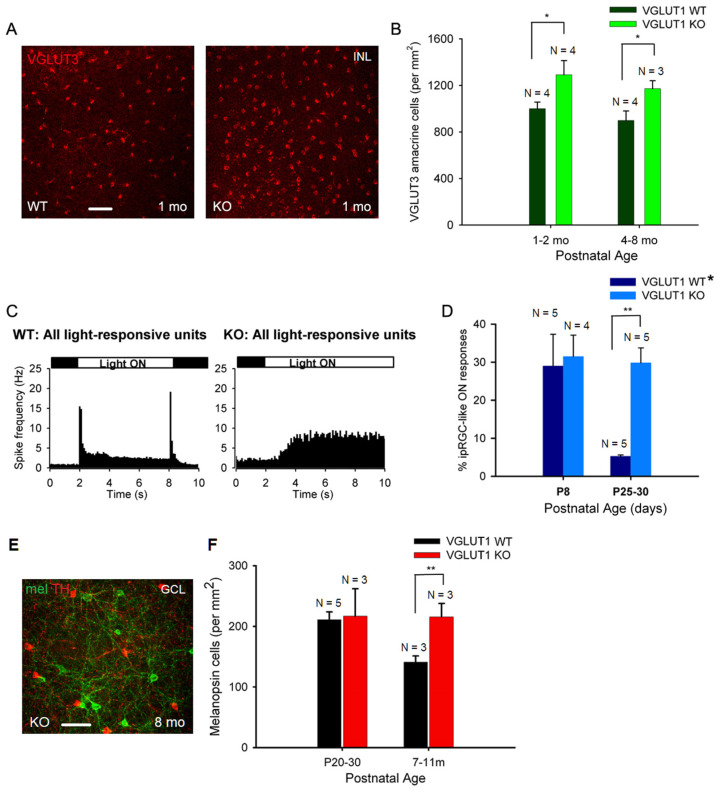
**Density of VGLUT3 amacrine cells and melanopsin ipRGCs is higher in older VGLUT1 null retina.** (**A**) VGLUT3 positive cell bodies in the INL viewed in the whole mounts of WT and VGLUT1 null (KO) retinas. More VGLUT3 cell bodies can be seen in VGLUT1 null retina. (**B**) The density of VGLUT3 cells was calculated in juvenile adult and adult/aged retinas. A significantly higher number of VGLUT3 amacrine cells was observed in VGLUT1 null retina in both age groups (* indicates *p* < 0.05). (**C**) Light responses to full field white light were measured from juvenile WT and VGLUT1 null retina using a multielectrode array. Peristimulus histogram of WT retina (left) exhibits fast ON and OFF responses with a latency of 100 ms, characteristic of rod and cone responses. VGLUT1 null retina (right) exhibits slow ON responses, with a latency of 1 sec or longer, which is characteristic of melanopsin ipRGCs. (**D**) Percentage of intrinsically light responsive units at P8, when rod cone mediated light responses are absent, is compared with juveniles that normally show very few ipRGC like units in WT, when rod/cone mediated responses are pharmacologically blocked [34]. Percentage of ipRGC like units is comparable between WT and VGLUT1 null retina at P8, but several folds higher in VGLUT1 null retina (** indicates *p* < 0.01). The WT* data are generated by combining C57Bl/6 and VGLUT1 WT/Het littermate data. (**E**) Melanopsin and tyrosine hydroxylase (TH) immunoreactivity in adult VGLUT1 null retina. (**F**) Counts of melanopsin cells in the GCL of juvenile and aged mice. This count is significantly higher only for aged VGLUT1 null mice (** indicates *p* < 0.01). Scale bar: 50 μm.

**Figure 7:**
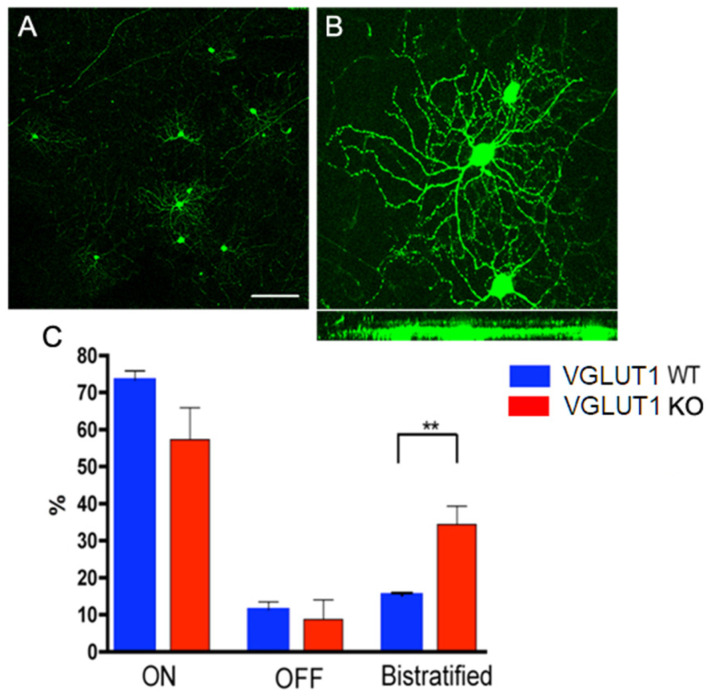
**Developmental maturation of RGC dendrites is incomplete in the VGLUT1 null retina.** (**A**) Thy1-YFP labeled RGCs at a lower magnification. RGCs are classified as ON, OFF, or ON/OFF based on their stratification patterns in the IPL. (**B**) Top panel shows whole mounted view of cell bodies and dendritic arbors of two ON RGCs (right) and one OFF RGC (left) marked with Thy1- YFP. Bottom panel shows the same YFP positive RGCs in Y/Z coordinates derived from z section stacks of confocal images. (**C**) Percentages of ON, OFF, and ON-OFF classes of RGCs in WT and VGLUT1 null (KO) retina. There is a significantly greater percentage of bistratified ON-OFF RGCs in the VGLUT1 null retina than in the WT retina (** indicates *p* < 0.01, *n* = 5 for each of WT and VGLUT1 null retina). The percentages of each class of RGCs in the VGLUT1 null retina resemble those in WT retina prior to eye opening. Scale bar: 100 μm for (**A**) and 50 μm for (**B**). The brightness of the image in B has been enhanced offline to visualize the dendritic arbors of the retinal ganglion cell, making the cell body overexposed.

**Figure 8:**
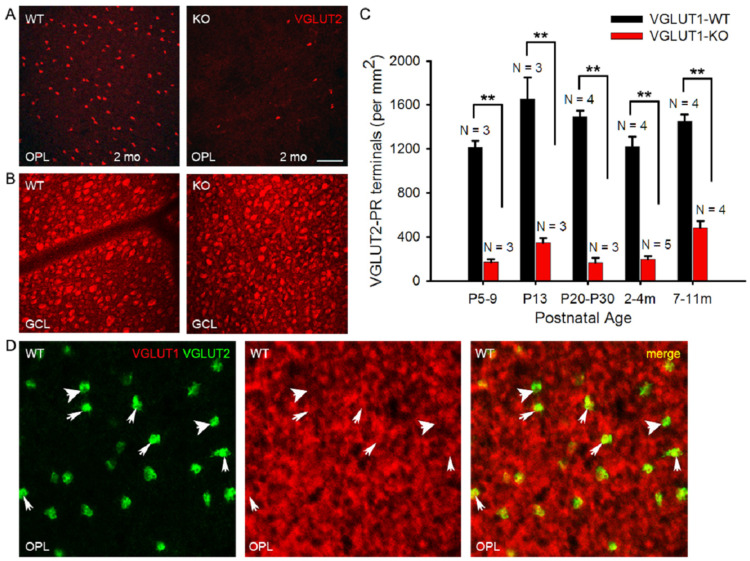
**Density of VGLUT2 photoreceptor pedicles is reduced at all ages in VGLUT1 null retina.** (**A**) **VGLUT2 immunoreactive cone pedicles in retinal whole mounts of 2 month-old WT and VGLUT1 null (KO) littermates, pictured at the OPL.** (**B**) The same retinas are viewed in the GCL for comparison. (**C**) Density of VGLUT2 cones in VGLUT1 null retina compared to WT littermates. There is a 2–4 fold reduction in the total number of VGLUT2 cone pedicles in VGLUT1 null mice at all ages, with a relative increase in aged null retina. For each retina, VGLUT2-immunoreactive pedicles were counted at two different near-central random positions and averaged. (**D**) Whole mounted C57Bl/6 (WT) retina is immunostained against VGLUT1 and VGLUT2, and the OPL is visualized at a higher magnification. Thick arrows show VGLUT2 positive terminals that are not colocalized with VGLUT1, while thin arrows mark cone pedicles positive for both VGLUT1 and VGLUT2. No statistics could be calculated for VGLUT1 and VGLUT2 colocalization because of the very high density of VGLUT1 terminals. Scale bar: 50 μm in (**A**,**B**) and 15 μm in (**D**). ** indicates *p* < 0.01. A 318.5 μm × 318.5 μm area or a 112.5 × 112.5 μm area was chosen for counting VGLUT2 pedicles in 2 separate near-central regions in each whole mounted retina and averaged.

**Figure 9:**
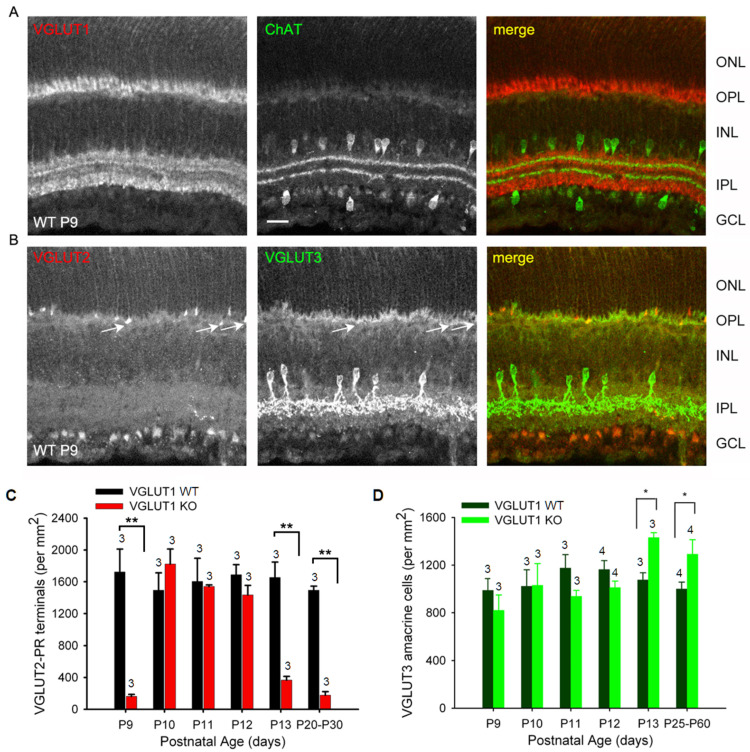
**Dynamic expression of VGLUT2 in the early postnatal VGLUT1 null retina.** Glutamatergic waves commence around P9 in the early postnatal developing retina. At this age, all 3 VGLUTs are detected in the retina immunohistochemically. (**A**) Expression of VGLUT1 and ChAT in the WT P9 retina. (**B**) VGLUT2 and VGLUT3 expression in the WT P9 retina. Arrows indicate VGLUT2 photoreceptor terminals also showing some VGLUT3 expression. VGLUT3 is not expressed in developed photoreceptors. (**C**) Density of VGLUT2 photoreceptor terminals at ages P9–P13 and in juvenile adult WT and VGLUT1 null retina, measured in the OPL of retinal whole mounts. (**D**) Density of VGLUT3 amacrines at ages P9–P13 and in juvenile adult WT and VGLUT1 null retina, measured in the INL of retinal whole mounts. Scale bar = 20 μm. * indicates *p* < 0.05, ** indicates *p* < 0.01. A 318.5 μm × 318.5 μm area or a 112.5 μm × 112.5 μm area was chosen for counting VGLUT2 pedicles in 2 separate near-central regions in each whole mounted retina and averaged. The number of animals (N) used are mentioned on each bar.

**Figure 10:**
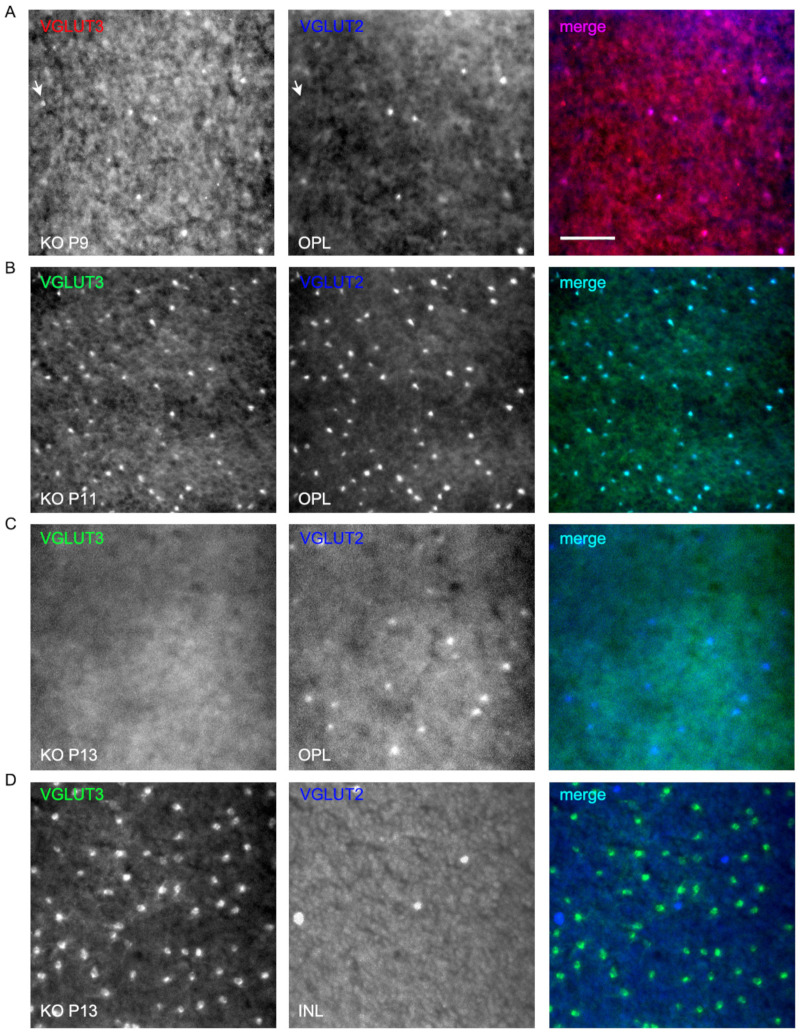
**Transient expression of VGLUT3 in cone pedicles of neonatal developing VGLUT1 null retina.** VGLUT3 expression is detected in the OPL of neonatal developing whole mounted retina in both VGLUT1 Het (Figure S5) and null littermates. We tried two different VGLUT3 primary antibodies, produced in rabbits and guinea pigs, and two different secondary antibodies, Alexa 568 and Alexa 488, to confirm this transient expression. VGLUT2 was stained with Alexa 647 to make sure VGLUT3 and VGLUT2 channels were spectrally separated from each other and one did not bleed into another. (**A**) VGLUT3 stained with Alexa 568 and VGLUT2 with Alexa 647 at P9 in the OPL of VGLUT1 null retina. Arrow points to a bright VGLUT3 photoreceptor that did not coexpress VGLUT2. (**B**,**C**) VGLUT3 stained with Alexa 488 and VGLUT2 with Alexa 647 in P11 and P13VGLUT1 null retina. At P11, VGLUT2 expression transiently increases in photoreceptors, and coincides with the transient expression of VGLUT3 in them. By P13, strong VGLUT3 expression is gone from cone pedicles, and the density of VGLUT2 expressing pedicles is reduced to the P9 level. (**D**) The same P13 retinas in C are visualized at the INL. VGLUT3 amacrine cells and some displaced VGLUT2 ganglion cells are seen. Scale bar: 50 μm.

**Figure 11:**
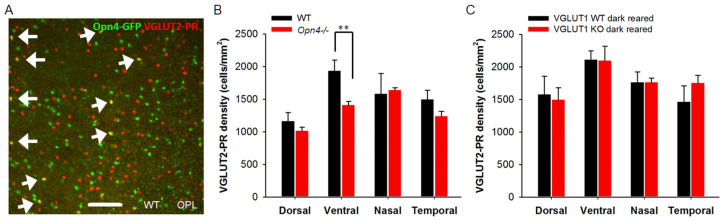
**Melanopsin ipRGCs influence expression of VGLUT2 in photoreceptor presynaptic terminals.** (**A**) Germline melanopsin-GFP mice express GFP in all cells with a history of *Opn4* gene expression. These mice show GFP expression in many cone pedicles, indicating that ipRGCs and Opn4-GFP positive photoreceptors were born from the same Opn4 expressing retinal progenitors. A minority of these GFP expressing photoreceptors express VGLUT2 in the adult WT mice. Here, the whole mounted retina of an adult Opn4-GFP; VGLUT1 WT mouse is immunostained with VGLUT2 and imaged at the OPL. The arrows indicate VGLUT2 cone pedicles expressing Opn4-GFP. (**B**) In melanopsin WT or Het mice, there is a moderate dorso-ventral gradient of VGLUT2 positive terminals. VGLUT2 pedicles are significantly lower in density in the ventral retina of melanopsin null mice (** indicates *p* < 0.01). (**C**) Dark rearing of VGLUT1 null mice until P15–22 restores the expression of VGLUT2 in photoreceptors to its normal density. A 318.5 μm × 318.5 μm area or a 112.5 μm × 112.5 μm area was chosen for counting VGLUT2 pedicles in 2 separate near-central regions in each whole mounted retina and averaged. Number of animals for B are WT: 3 and *Opn4^−/−^*: 4 and C are WT: 4 and KO: 3.

Like in humans, mouse retina is rod-dominated [37,38]. Cone pedicles are approximately 30 times less numerous compared to rod spherules and are countable in the OPL of whole mounted retina (Figure 3C). A small subset of cone pedicles is immunoreactive for both VGLUT1 and VGLUT2 in the WT mouse retina [7]. They are also immunoreactive for cone specific signaling protein cone arrestin (Figure 3C). The density of both cone arrestin and VGLUT2 positive pedicles is found to be significantly lower in the aged VGLUT1 null retina compared to the littermate control, when examined in the OPL of retinal whole mounts (Figure 3C). The quantification of cone arrestin pedicles in the OPL of central retina reveals that cone pedicles are fewer in the P13 VGLUT1 null retina but reach WT density by P22, indicating an initial delay in the functional activation of these photoreceptors in VGLUT1 null retina (Figure 3D). Their density progressively reduces with age, ultimately becoming almost half as numerous as the adult WT cones. In individual cones of VGLUT1 null retina, the cone arrestin signal is lost from the OS first, followed by the pedicles. At 7 months of age, a majority of cone outer segments of VGLUT1 null retina lost the cone arrestin signal, yet about 50% of all cone pedicles had detectable expression of cone arrestin. The extent of loss of cone arrestin in the OPL varied between retinal areas. We counted pedicles only in the whole mounted central retina. Since the loss of rhodopsin protein from the OS of VGLUT1 null retina is nearly complete by 4 months of age (Figure 2A), we pulled all data for 4 months and beyond as adult or aged category in which photoreceptor degeneration had already occurred, and presented in Figure 3D and subsequent statistics in Figure 4, Figure 5, Figure 6, Figure 7, Figure 8, Figure 9, Figure 10 and Figure 11. Data presented in Figure 2 and Figure 3 suggest that maintained VGLUT1 mediated glutamatergic excitatory neurotransmission by photoreceptors is required for their own survival and health in the WT. The marked loss in VGLUT2 positive cone pedicles in VGLUT1 null retina is discussed later in Figure 8.

### 3.2. Loss of Glutamatergic Neurotransmission in the OPL Triggers Synaptic Disorganization and Ectopic Synaptic Formations in the ONL

A lack of b-wave in the VGLUT1 null retina [12] and age related progressive loss of photoreceptor markers in these mice (Figure 2 and Figure 3) suggest possibilities of age related reduction in the synaptic formation at the photoreceptor to bipolar cell contacts in the OPL. CtBP2, a member of the ribbon synaptic complex at the photoreceptor to bipolar and bipolar to ganglion cell excitatory presynaptic sites, shows punctate expression in the OPL of WT and VGLUT1 null retina. The simplified schematic in Figure 4A shows the organization of photoreceptor ribbon triads, formed between one presynaptic rod spherule, one postsynaptic rod bipolar, and two postsynaptic horizontal cell processes. Postsynaptic G-protein coupled metabotropic glutamate receptor 6 (mGLUR6) is also shown in the schematic. Similar synaptic formations are also formed at the cone pedicles in the OPL (not shown here). Figure 4A inset shows the OPL of an adult WT retina, immunostained for CtBP2, PKCα expressing rod bipolar cells, and mGLUR6, expressed at the dendritic tips of all ON bipolar cells. On closer examination, few unusual CtBP2 and mGLUR6 puncta were located in the outer nuclear layer (ONL) of older adult VGLUT1 null retina (Figure 4B), a place where no ribbon synaptic formations are typically detected in the WT retina. The CtBP2 and mGLUR6 puncta are closely associated with each other at those ectopic synaptic sites, indicating rudimentary ribbon synaptic assembly in the ONL of these mice. Concurrent to that, elongation of a few rod bipolar and horizontal cell dendrites into the ONL of VGLUT1 null retina was detected, indicating the morphological disorganization of the OPL (Figure 4B,C). The molecular events underlying the horizontal and rod bipolar cell ectopic dendritic projections into the ONL are still not understood. The dendritic tips and shafts of these ectopic projections are often associated with CtBP2 and mGLUR6 puncta, suggesting ectopic ribbon triad formation with the photoreceptor cell bodies or axonal shafts. It is not clear whether these ectopic synapses are functional and if they contribute in resisting the progressive degeneration of the photoreceptors. These ectopic synaptic formations do not show any regular tiling of the ONL. Rod bipolar and horizontal dendritic protrusions in the ONL also do not exhibit any regular patterns of organization. These misplaced dendritic brunches elongate with age, starting at developed young adulthood (2–3 months). Horizontal processes start sprouting in the ONL first, followed by rod bipolar cells, as if they react to an attractant in the ONL, or leave the OPL due to a loss of an adherent of unknown nature (data not shown). More and more rod bipolar and horizontal cell dendrites leave the OPL and project into the ONL as the VGLUT1 null mice age. Unlike rod bipolar cells, cone bipolar cells seldom send out dendrites in the ONL (data not shown). The loss of VGLUT1 does not disrupt the expression of mGLUR6, but the expression of some ionotropic glutamate receptors (for example, kainite receptor GluR5 and AMPA receptor GluA1) at the OFF bipolar cell flat contacts is reduced (Figure S1). The molecular events leading to the sustenance of mGLUR6 expression even in the absence of any synaptic input are not known yet.

### 3.3. In Contrast to Progressive Decrease in Photoreceptor Densities, Bipolar Cells, VGLUT3 Amacrine Cells, and Melanopsin ipRGCs Have Higher Densities in Juvenile, Adult, and Aged VGLUT1 Null Retina

Among the inner retinal neurons, rod bipolar and glycinergic amacrine cells are the last to differentiate from retinal progenitors. These cells are born postnatally, P0 onwards, during the time VGLUT1 starts to express at the ribbon presynaptic terminals in the OPL and IPL [16]. We wanted to examine whether the vesicular glutamate release in the OPL and IPL influences the histogenesis of these cell types, in contrast to prenatally born VGLUT2 expressing retinal ganglion cells, rods, cones, and GABAergic amacrine and horizontal cells. CtBP2 puncta seemed relatively more numerous in the IPL of VGLUT1 null retina, when examined in radial sections at a lower magnification (Figure S2A), indicating a paradoxical increase in the number of ribbon synapses in the IPL in the absence of vesicular glutamate release at the ribbon synapses. We hypothesized that there were more ribbon synaptic zones per presynaptic terminal or more number of presynaptic cells in general. In line with this hypothesis, PKCα positive cell bodies seemed more numerous in the radial sections (Figure 5A) and in the whole mounted retina (not shown) of juvenile developing VGLUT1 null mice, compared to the WT littermate control. Rod bipolar cells are too numerous to be counted in the whole mount. We measured the relative fluorescence of PKCα immunoreactivity in the INL of whole mounted adult VGLUT1 null retina and found a significant increase in the mean fluorescence, compared to WT from the same age group (Figure S2B). The visible PKCα cell bodies on the radial sections were counted in the INL at juvenile developing, developed young, and older adult ages. We found a significant increase in the rod bipolar cell numbers at all ages (Figure 5B). L7/pcp promoter is specifically activated in both rod and ON cone bipolar cells. We raised VGLUT1 null mice in the L7-EGFP background and tested the EGFP expression in VGLUT1 null retinas at juvenile developing, developed young, and older adult groups. At all ages, L7-EGFP expressing cells seemed more numerous in VGLUT1 null retinas compared to age matched WT control. When radial sections of L7-EGFP retinas were immunostained with PKCα, they overlapped well, except for some L7-EGFP cells that were PKCα negative (Figure 5C,D). Those cells were perceived as ON cone bipolar cells. Their density also seemed relatively higher in the inner part of the INL of VGLUT1 null retina, although they could not be reliably counted. A similar staining pattern was observed when WT and VGLUT1 null retina were immunostained with G_o_α, another ON bipolar cell marker (data not shown). In summary, there seemed to be a significant increase in the rod and ON cone bipolar cells. In contrast, there was no change in calbindin positive horizontal cells, cholinergic cholineacetyl transferase (ChAT) positive starburst amacrine cells, and dopaminergic tyrosine hydroxylase (TH) positive amacrine cells, when counted in the radial sections or whole mounted retinas of VGLUT1 null mice and compared with the age matched control (data not shown). These interneurons are GABAergic and are born prenatally in the retina; their histogenesis may not be dependent on VGLUT1 mediated glutamatergic extrinsic signaling [16].

We next examined the cellular densities of VGLUT3 positive glycinergic amacrine cells. These cells respond to small object motion. They use both glutamate and glycine to modulate ON and ON-OFF direction selective ganglion cells and W3 amacrine cells [39]. These cells are a source of vesicular glutamate release in the IPL in the absence of VGLUT1 mediated vesicular glutamate release from bipolar cells. Another potential candidate for vesicular glutamate release in the IPL is melanopsin ipRGCs. They express VGLUT2, and release glutamate at their presynaptic zones on their axonal collaterals to activate dopaminergic amacrine cells in the IPL [40]. There is no evidence of other VGLUT2 positive retinal ganglion cells releasing glutamate in the retina. Figure 6A shows VGLUT3 staining in the INL of whole mounted WT and VGLUT1 null retinas. Their densities seemed moderately higher in juvenile developing, developed young, and older adult VGLUT1 null retinas compared to the age matched controls (*p* < 0.05, Figure 6B). Retinal ganglion cells were immunostained with VGLUT2 and visualized in the GCL of whole mounted retinas. The cell density was not conducive for counting individual cells. The relative mean fluorescence intensity of VGLUT2 in the GCL of whole mounted adult VGLUT1 null retina was significantly higher, because of either the higher expression of VGLUT2 in individual ganglion cells, or their higher cell densities, the latter being the more plausible reason, asserted by visual inspection (Figure 8B and Figure S3).

We employed multielectrode array (MEA) recording to study the density of ipRGCs. We recorded action potential firing patterns in the GCL of whole mounted retina in presence of a 6 s long full field white light stimulus. In juvenile developing WT retina, sustained and transient ON and OFF responses can be recorded at P13 and later. After kmeans clustering of light responses, 100 or more individual units (presumably cells) were isolated and classified as ON or OFF cells, based on whether their action potential firing increased when light was switched ON, versus when light was switched OFF (Figure 6C). Light stimuli elicited slow sustained ON responses with a characteristic latency of 1 s or longer, typical for ipRGCs, in the VGLUT1 null retina (Figure 6C). We used pharmacology to isolate ipRGC like responses in WT retina, as previously described [34]. We recorded light responses in the WT retina in the presence of antagonists that blocked nicotinic acetylcholine, AMPA, Kainate, NMDA, and mGLUR6 receptors. This condition caused a total synaptic block in the retina, leaving intrinsically photosensitive light responses. We counted all units as ipRGCs that showed slow ON responses in the presence of synaptic block in WT and VGLUT1 null retinas (Figure 6D). The density of ipRGCs in WT and VGLUT1 null retina is comparable at P8, an age when rod/cone mediated light responses are absent, but many folds higher in the juvenile developing ages. Immunostaining for melanopsin cells is another way of marking ipRGCs (Figure 6E). Unfortunately, our melanopsin antibody did not stain all ipRGCs. It mostly stained M1 and M2 ipRGCs, out of five different ipRGCs described in the literature [41]. Their density was not higher in the juvenile developing age group but significantly higher in older adult VGLUT1 null retina compared to the age matched WT (Figure 6F). In summary, all glutamatergic neurons in the inner retina are present at higher densities in the aged VGLUT1 null retina, in contrast to rods and cones that are fewer in the aged VGLUT1 null retina compared to the age matched controls.

### 3.4. Postnatal Refinement of RGC Dendritic Arbors Is Delayed in VGLUT1 Null Mice

The developmental cascades in VGLUT1 null retina are incomplete at P25–P30, at an age when retinal pathways are fully developed in mice and cortical maturation is underway [42]. We could detect infrequent cholinergic waves at P25, with a frequency of approximately 0.5/min that became more frequent when inhibition was suppressed with GABA and glycine receptor blockers, and could be blocked by nicotinic acetylcholine receptor blocker DHβE (Figure S4). We hypothesized that the dendritic pruning of retinal cells would also be incomplete at P30. The majority of retinal ganglion cells in the first postnatal week diffusely stratify in both the ON and OFF sublamina of the IPL. By 4 weeks of age, the dendritic arbors of a majority of them become monostratified in either the ON or OFF sublamina. Dark rearing retards this refinement of retinal ganglion cells dendritic arbors, consistent with the idea that visual input to rod/cone/melanopsin mediated pathways is required for the dendritic maturation of retinal ganglion cells [43,44]. We tested whether a reduced vesicular glutamate release in the IPL of VGLUT1 null mice influenced the refinement of retinal ganglion cells dendritic arbors. Following the approach of Tian and Copenhagen [43], we studied Thy1-YFP mice, which expressed YFP in a sparse sample of most types of retinal ganglion cells (Figure 7A,B). VGLUT1 null mice expressing Thy1-YFP were compared to WT Thy1-YFP littermates. Hundreds of retinal ganglion cells were imaged and grouped based on their stratification patterns. The percentages of retinal ganglion cells with monostratified dendrites (ON or OFF sublamina) and bistratified dendrites (ON and OFF sublamina) are plotted in Figure 7C. Significantly more retinal ganglion cells displayed bistratified or diffusely stratified dendritic arbors in the VGLUT1 null retina compared to their WT littermates at ~P30, indicating that there are many underpruned and immature retinal ganglion cells in the VGLUT1 null mice at the juvenile developed age. These data are in striking contrast to the data presented in Figure 3D, where we showed that cone arrestin positive cone pedicles reached WT like density in VGLUT1 null retina by P22, indicative of completed histogenesis and pruning of photoreceptors by that age. Retinal ganglion cell dendritic pruning study was not extended to older ages. The prediction was that some retinal ganglion cells would always have diffused immature arbors in VGLUT1 null mice, given that their density always remained elevated, and excess cells never got pruned away to match the WT density (see Figure S3). Both the pruning of retinal ganglion cell dendrites and pruning of excess cells are presumably suboptimal in the absence of sufficient vesicular glutamate release. These, together with the previous section, establish a profound phenotypic effect of germline deletion of VGLUT1 on postnatal differentiation and maturation of glutamatergic inner retinal neurons: bipolar, amacrine, and retinal ganglion cells.

### 3.5. VGLUT2 Positive Cones Are Fewer at All Ages in the VGLUT1 Null Retina

We show in Figure 3C that VGLUT2 positive cone photoreceptor pedicles are fewer in the older adult VGLUT1 null retina compared to the littermate control. VGLUT2 immunoreactivity is associated with about 10% of all cone pedicles. VGLUT2 positive cones coexpress VGLUT1, are distributed in a regular array in the OPL indicating they are a distinct type of photoreceptors, and show no obvious dorso-ventral gradient or S-opsin specificity, unlike true blue cones [7]. They may drive vesicular glutamate release in the OPL of VGLUT1 null retina. Unlike cone arrestin expression that reaches the WT level by P22 in VGLUT1 null retina, VGLUT2 cone pedicle density is significantly low in VGLUT1 null retina at all ages, starting from P5 when they start to express VGLUT2 at the cone pedicles in these mice (Figure 8A,C). We checked the GCL of the same VGLUT1 null retinas to confirm that the diminished staining of VGLUT2 in the OPL was not a consequence of poor antibody reaction. VGLUT1 null retina had significantly more VGLUT2 positive ganglion cells in the GCL compared to the littermate control (Figure S3 and Figure 8B). The VGLUT2 expression pattern in cone pedicles of VGLUT1 null retina is atypical of what we reported for other photoreceptors in Figure 2 and Figure 3: we showed a lack of activity related photoreceptor degeneration with age, happening at a faster rate compared to WT. We hypothesized that there were two types of VGLUT2 cones, one that expressed VGLUT1 and VGLUT2 and other that expressed VGLUT2 alone. The former type did not express VGLUT2 in the absence of VGLUT1 coexpression in the VGLUT1 null retina. To test this hypothesis, we immunostained adult C57Bl/6J retinal whole mounts with VGLUT1 and VGLUT2. We could find a few VGLUT2 positive pedicles in single sections that had very little VGLUT1 co expression, if any (Figure 8D). Yet, it is difficult to prove if these few VGLUT2-only cones were selectively born and survived in the VGLUT1 null retina.

### 3.6. VGLUT2 and VGLUT3 Expression Changes Dynamically During the First 2 Weeks of Postnatal Development

As we discussed before, VGLUT1 null retina did not have propagating glutamatergic waves, typically evidenced in the P10–12 postnatal ages in WT retina [20]. In that period, the pruning of newborn retinal cells takes place; the waves of apoptotic cell deaths sweep the retina, removing excess cells and redundant synaptic connections [45,46]. Studies in chick retina provided evidence that signaling cascades involving extrinsic ascorbic acid and glutamate and intrinsic nitric oxide could modulate CREB phosphorylation, thereby influencing the decision between cell survival and apoptotic cell death [17]. Altered densities of glutamatergic cells in the VGLUT1 null retina, outlined in the previous sections, is indicative of a role of VGLUT1 mediated glutamate release in the inner retinal histogenesis and, hence, changes in the rate of cell survival. At P9, VGLUT1 as well as VGLUT2 and VGLUT3 are expressed in the WT retina (Figure 9A,B). In VGLUT1 null retina, cholinergic waves are seen in this period, and continue to remain until P25, although with a lower frequency than normally seen in P4–P8 stages (Figure S4 [47]). At P9–P13, we noticed the transient expression of VGLUT3 in the OPL, mostly in photoreceptor terminals and some bipolar and horizontal cell bodies, confirmed by double immunostaining for VGLUT3 and cell specific markers (data not shown). This expression fades away by P22, and in juvenile adults, VGLUT3 is selectively expressed in one type of glycinergic amacrine cells, as discussed in Figure 6.

We followed the expression of bipolar, VGLUT3 amacrines, VGLUT2 cones, ganglion cells, and melanopsin cells on a daily basis during P9–P13 and noticed differences between WT and VGLUT1 null retina. VGLUT2 photoreceptor counts changed dynamically in VGLUT1 null retina, increasing to the WT level by P10 and decreasing abruptly after P12 to an approximately 4 fold lower density (Figure 9C). In contrast, PKCα positive rod bipolar cell densities seemed highly variable and not countable (not shown), and the density of VGLUT3 amacrine cells remained more or less constant until P12 and then increased moderately in the VGLUT1 null retina (Figure 9D, also Figure 6B). Densities of melanopsin and VGLUT2 immunoreactive ganglion cells or ipRGCs recorded on MEA in the VGLUT1 null retina did not differ from the WT littermate control at P9–P13 (data not shown). Evidently, transient VGLUT3 expression is selectively strongest in VGLUT2 cones during this dynamic expression period, both in WT (Figure S5) and VGLUT1 null retina (Figure 10, also arrows in Figure 9B). Whereas transient expression of VGLUT3 does not influence the density of VGLUT2 cones in the OPL of WT, it coincides with the transient increase in VGLUT2 expression in the cone pedicles of VGLUT1 null retina. We conclude that a subset of VGLUT2 cones requires the coexpression of VGLUT1 for expressing VGLUT2. In the absence of VGLUT1, the transient expression of VGLUT3 in these photoreceptors is sufficient to turn VGLUT2 expression on transiently. To confirm that VGLUT3 impacts the expression of VGLUT2 in cones, we studied the density of VGLUT2 cones in three VGLUT3 Het and null retinas within the juvenile developing age group (P13–P45, gift from Rebecca Seal, 11). The VGLUT2 cone pedicles have doubled in density in the VGLUT3 null retina at juvenile ages (Figure S6), suggesting that VGLUT3 may also impact glutamatergic gene expression and histogenesis.

### 3.7. Light Activation of ipRGCs Suppresses VGLUT2 Expression in Cone Pedicles of VGLUT1 Null Retina

Recent research on melanopsin ganglion cell’s role in early retinal development suggests that melanopsin ipRGCs send their axonal collaterals to the OPL and IPL, and presumably influence synapse formation [48]. They drive oxytocin dependent synaptogenesis in the hypothalamus [49]. They also impact the histogenesis of rods [50]. Melanopsin-GFP transgenic mice show GFP expression in a minority of cone pedicles (Figure 11A). These cones may have differentiated prenatally from the same retinal progenitors that gave birth to melanopsin ganglion cells. A few but not all of these melanopsin-GFP cone pedicles express VGLUT2 (arrows in Figure 11A). We examined VGLUT2 cone density in the melanopsin null retina, and found a significant reduction in the density of these photoreceptors in the ventral retina (Figure 11B). Interestingly, in the melanopsin WT/Het littermates, there is a moderate dorso-ventral gradient for VGLUT2 photoreceptor, unlike in C57Bl/6 WT and VGLUT1 WT or null retina ([7] our data). Since melanopsin ipRGCs are the only photosensitive cells with active downstream glutamatergic signaling pathway in the VGLUT1 null retina [12], we dark reared them to functionally inactivate melanopsin mediated light signals in the early pre and postnatal ages, and looked for changes in VGLUT2 cone density. Inactivating melanopsin cells by dark rearing between E15 and P25 reduced VGLUT2 expression deficits, bringing the density of VGLUT2 cones up to the WT level (Figure 11C). This result drives us to the conclusion that VGLUT2 expression in the majority of VGLUT2 cones is controlled by the cellular coexpression of VGLUT1 and VGLUT3 and retrograde glutamatergic input from melanopsin ipRGCs [48,50]. Finally, there are a small minority of VGLUT2 cones that express VGLUT2 alone.

## 4. Discussion

### 4.1. Age Related Photoreceptor Degeneration and Increase in Histogenesis of Inner Retinal Glutamatergic Neurons in VGLUT1 Null Mice

The lack of phototransduction or synaptic release of glutamate from photoreceptors induces age related retinal morphological changes (this study [35]). Previous studies in knockout mice carrying null or missense mutations in the retinal pigment epithelial or photoreceptor markers, for example, phototransduction related retinoid isomerohydrolase enzyme RPE65, Ca_v_1.4 voltage gated calcium channel necessary for vesicular release of glutamate (*Cacna1f*, *Cacna2d4*) and its regulator calcium binding protein CaBP4, ribbon structural protein Piccolo, Bassoon, and RIBEYE, showed that silencing of photoreceptors causes age related photoreceptor degeneration [35,51]. In some models, cones die earlier than rods, although it is more common to see rods dying earlier than cones. In many of these transgenic models, horizontal and rod bipolar cells send out sprouting dendrites in the ONL at adult ages, a behavior normally seen at early postnatal developing age, as part of the pruning process, when dendrites of horizontal cells extend to the ONL and INL before finally ramifying narrowly in the OPL and invaginating into ribbon hotspots on rod spherules or cone pedicles [35]. Germline deletion of VGLUT1 protein phenocopies these effects, as demonstrated in Figure 1, Figure 2, Figure 3 and Figure 4. Even without any mutation in photoreceptor structure and function related genes, slow age related photoreceptor degeneration and shrinkage of the retina take place [36]. Silencing of rods and cones accelerates this process.

In addition to the age related loss of rod and cone markers, indicative of photoreceptor dystrophy, we observed a sustained increase in inner retinal glutamatergic cells at juvenile and adult ages in VGLUT1 null retina (Figure 5 and Figure 6). Histogenesis of cells is a two-step process, involving cellular differentiation by switching to cell intrinsic mechanism, governed by a change in extrinsic signaling molecules and mitogenic blockers, followed by the pruning of extra cells and their immature dendrites, mostly governed by presynaptic input [16]. Our findings suggest that either the number of retinal progenitors exiting cell cycle and adopting the bipolar, VGLUT3 positive amacrine or VGLUT2 positive ganglion cell fate or the choice between survival and apoptosis of all newly born glutamatergic neurons is shifted in the absence of synaptic or extrasynaptic VGLUT1 mediated glutamate release. This is surprising because cones, retinal ganglion cells, and a majority of rods are born prenatally, before VGLUT1 starts to express in the OPL and IPL [1,5,16]. In the late prenatal and early postnatal developing stages, E18 onwards, both VGLUT1 and VGLUT2 staining show very high background in the retinal progenitors in WT. This background could not be reduced below the fluorescence detection level by diluting the antibodies to a suboptimal concentration [Majumdar, unpublished information]. Therefore, it is hard to rule out VGLUT1’s intracellular role in prenatal retina, after observing a paradoxical increase in the number of retinal ganglion cells. An alternative to this hypothesis is the dysregulation of cell fate choice of retinal progenitors giving birth to all retinal glutamatergic cells postnatally in VGLUT1 null retina. It is possible that some of the late born glutamatergic cells adopt retinal ganglion cell fate instead of bipolar or VGLUT3 positive amacrines in VGLUT1 null retina. Bipolar and amacrine cells differentiate out from post-mitotic retinal progenitors at P0–P7, during the time VGLUT1 concentrates at the photoreceptor and bipolar cell axon terminals, prior to ribbon synapse activation and start of glutamatergic wave at P10 [1,5,12,16,20]. In this time frame, pruning of excess cells take place, as the synaptic connections mature. Apoptotic cell death of retinal ganglion cells happens constantly between P0 and P22 with peak death happening at P2 and P15 [45]. About 2–3% retinal ganglion cells die every day in this period. A similar apoptotic death of bipolar and amacrine cells happens between P8 and P17 with the peak of death at P9–P10. The fact that a higher density of all these cells is registered in VGLUT1 null mice around P30 makes it most prudent to hypothesize that apoptotic cell death of glutamatergic cells is slowed down in VGLUT1 null retina. This would suggest that VGLUT1 mediated glutamate release control choice between cell survival and death in postnatal retina [17]. Yet, it is hard to rule out the possibility of reduced glutamate release affecting the percentage of glutamatergic cells exiting cell cycle. Birth of rods and cones was not impacted in VGLUT1 null retina. Histogenesis of specifically postsynaptic glutamatergic neurons was impacted. Glutamate is in general excitotoxic [1]. Reduced glutamate in the synaptic clefts may cause reduced cell death. This corollary should apply to all retinal neurons, and not just the postsynaptic glutamatergic cells. Since we see a specific increase in the inner retinal glutamatergic cell numbers, we can safely rule out reduced excitotoxicity playing a role in histogenesis of glutamatergic cells. More detailed biochemical and ultrastructural imaging analysis of VGLUT1 null retina in comparison to age matched WT can clear our understanding and help us choose between so many competing hypotheses.

### 4.2. Dynamic Expression of VGLUT2 in Some Cones in VGLUT1 Null Retina Is Governed by Transient Expression of VGLUT3 in These Cones and Can Be Influenced by Melanopsin ipRGCs

The developmental expression pattern of VGLUTs in the retina is similar to the pattern seen in the brain [5,9]. VGLUT2 is expressed in newly born retinal ganglion cells prenatally, yet their necessity in the prenatal retinal development is debated. VGLUT1 is the main vesicular transporter required for image forming vision [12]. About 10% of all cones express VGLUT1 and VGLUT2. Overall, 70–80% of these VGLUT2 cones lose VGLUT2 expression in the absence of VGLUT1 coexpression in the VGLUT1 null retina (Figure 8). The remaining VGLUT2-only cones may be functional. They express CtBP2 and ramify in the OPL at all ages; no VGLUT2 cone terminals retract to the ONL (data not shown). These surviving VGLUT2 cones did not have a regular mosaic and did not show bias for S-opsin or L-opsin (data not shown). Therefore, they are not a special type of cones and are not different compared to the VGLUT2 cones described by Waessle and colleagues in the WT retina [7]. VGLUT1 knockdown with shRNA in developing neocortical neurons in culture suppressed synaptic vesicle marker expression at synapses that normally coexpress VGLUT1 and VGLUT2 [52]. This is similar to our findings outlined in Figure 8. VGLUT2 only cones in the VGLUT1 null retina are too few to be studied for their physiological function. VGLUT1 and VGLUT2 coexpressing presynaptic terminals often have single synaptic vesicles containing both the transporters. Physiological studies in the calyx of held showed that VGLUT1 expression increased the speed of vesicle filling and release, when co expressed with VGLUT2 [15]. Therefore, we speculate that, in the absence of VGLUT1, VGLUT2 only cones may not be capable of fast neurotransmission image forming vision requires.

We show that VGLUT3 transiently expresses in the OPL and IPL and in the cell bodies of some horizontal and bipolar cells (Figure 9 and Figure 10) of both WT and VGLUT1 null retina. Its expression is strongest in VGLUT3 amacrine cells at all ages and in VGLUT2 positive cones around P10–P11. Between P9 and P13, both VGLUT2 and VGLUT3 antibodies stain the OPL and IPL faintly. Yet, the present evidence suggests that they do not participate in synaptic or extrasynaptic glutamate release, since both image forming vision and glutamatergic waves are absent in VGLUT1 null retina [12,20]. Most astonishingly, VGLUT3 coexpression transiently restores VGLUT2 expression in the cones in VGLUT1 null retina around P11. These VGLUT2 cones normally coexpress VGLUT1 and VGLUT2 and stop expressing VGLUT2 in the absence of VGLUT1. Such dependence of one VGLUT expression on another may have to do with VGLUT2 variants. Some unidentified variants of VGLUT2 may require the coexpression of VGLUT1 or VGLUT3. Finally, the dark rearing of VGLUT1 null retina can also restore VGLUT2 expression in cones. Thus, retrograde input from melanopsin may also influence VGLUT2 expression. Another possibility is that phototransduction machinery within cones influence VGLUT2 expression. These hypotheses require further testing. In summary, intracellular, synaptic, or extrasynaptic VGLUT1 is required for differentiation of the majority of VGLUT2 cones and adopting their normal expression of VGLUT2 at their terminals in WT retina.

## 5. Conclusions

In this study, we outlined the phenotypic effect of genetic loss of VGLUT1 protein on histogenesis, differentiation, and pruning of glutamatergic neurons of mice retina. We have unequivocally established that vesicular glutamate release from photoreceptors in the OPL and from bipolar cells in the IPL influences the density of bipolar, VGLUT2 positive cones, VGLUT3 positive amacrines, and VGLUT2 positive retinal ganglion cells and the pruning of retinal ganglion cells dendrites, in addition to the loss of activity related accelerated photoreceptor degeneration in adult retina. Furthermore, the VGLUT2 expression in the cones can be dynamically altered by either the transient coexpression of VGLUT3 in VGLUT2 positive cones or the suppression of melanopsin ipRGC activity by dark rearing. In summary, our paper describes the main morphological differences observed in VGLUT1 null retina, in comparison to the WT retina.

## Figures and Tables

**Table 1:** List of primary antibodies used for immunohistochemistry.

Antigen	Antibody Dilution	Source Animal, Company
PKCα	1:500 [23]	Mouse, Santa Cruz Biotechnology, USA
PKCα	1:10,000 [24]	Rabbit, Sigma-Aldrich, USA
SV-2	1:5000 [25]	Mouse, Developmental Studies Hybridoma Bank, USA
Calbindin	1:2000 [24]	Rabbit, Millipore, USA
VGLUT1	1:1000 [5]	Rabbit, Synaptic Systems, Germany
VGLUT2	1:2000 [7]	Guinea pig, Millipore, USA
VGLUT2	1:500 [7]	Rabbit, Millipore, USA
VGLUT3	1:2000 [26]	Guinea pig, Millipore, USA
VGLUT3	1:1000 [8]	Rabbit, Millipore, USA
CtBP2	1:5000 [27]	Mouse, BD Transduction, USA
PKARIIβ	1:1000 [28]	Rabbit, BD Transduction, USA
mGLUR6	1:500 [29]	Guinea Pig, Neuromics, USA
GluK1 (GluR5)	1:100 [30]	Goat, Santa Cruz Biotechnologies, USA
Cone arrestin	1:1000 [31]	Rabbit, Millipore, USA
TH	1:400 [24]	Rabbit, Millipore, USA
ChAT	1:400 [32]	Goat, Millipore, USA
Melanopsin	1:2000 [33]	Rabbit, Millipore, USA

## Data Availability

The original contributions presented in this study are included in the article/Supplementary Material. Further inquiries can be directed to the corresponding author.

## Supplementary Materials

The following supporting information can be downloaded at: https://www.mdpi.com/article/10.3390/brainsci15091024/s1. Figure S1: Ionotropic glutamate receptor expression is diminished at OFF cone bipolar cell synapses; Figure S2: Expression of ribbon presynaptic protein CtBP2 and rod bipolar cell marker PKCα is higher in VGLUT1 null retina; Figure S3: Ganglion cell density has increased in the VGLUT1 null retina; Figure S4: Cholinergic wave in the juvenile VGLUT1 null retina; Figure S5: Transient expression of VGLUT3 in cone pedicles of neonatal developing VGLUT1 Het; Figure S6: Density of VGLUT2 cones is higher in juvenile/adult VGLUT3 null retina.
