# Supplementary material for: Genetic Loss of VGLUT1 Alters Histogenesis of Retinal Glutamatergic Cells and Reveals Dynamic Expression of VGLUT2 in Cones
Source: Brain Sci. 2025 Sep 22;15(9):1024. doi: 10.3390/brainsci15091024 (PMC12468242; doi:10.3390/brainsci15091024)
Supplement: Supplementary file 1 [file brainsci-15-01024-s001.zip › brainsci-3821288-supplementary.pdf]

Supplementary Material for

Genetic Loss of VGLUT1 Alters Histogenesis of Retinal Glutamatergic Cells and Reveals Dynamic Expression of VGLUT2 in Cones

Sriparna Majumdar [1,2,*,†] and Vincent Wu [3,†]

[1] Department of Biology, Stanford University School of Medicine, Stanford, CA 94305, USA
[2] Computer Science Department, City College of San Francisco, San Francisco, CA 94112, USA
[3] Kaiser Permanente Redwood City Medical Center, Redwood City, CA 94063, USA
* Correspondence: smajumda@mail.ccsf.edu
† Formerly: Department of Ophthalmology, University of California San Francisco School of Medicine, San Francisco, CA 94143, USA.

Figure S1:

Ionotropic glutamate receptor expression is diminished at cone to OFF bipolar synapses (A) Kainate receptor GluR5 expression in the OPL of VGLUT1 WT and null (KO) retina. The GluR5 expression is strongly reduced at the OFF bipolar cells basal contacts in VGLUT1 null retina. (Ba, Bb) Higher magnification view of the OPL stained with GluR5. Isolated hotspots were counted in radial sections of VGLUT1 WT and null retina between P19-P30. Occasionally, more than one hotspot appeared fused to each other. They were counted as a single composite hotspot. Each arrow indicates one such composite hotspot in single micrographs. (C) The density of GluR5 is significantly reduced in VGLUT1 null retina ($p < 0.01$). (D) GluR5 expression at the dendritic tips of PKARII positive type 3b OFF bipolar cells in 9 months old VGLUT1 null retina. Plenty of retracting PSD-95 positive photoreceptor terminals were seen in the ONL. The OFF bipolar dendrites seldom sprout and no postsynaptic GluR5 puncta is associated with retracting photoreceptor terminals in adult VGLUT1 null retina. Similar results were obtained when VGLUT1 null retina were stained with AMPA receptor GluA1. Scale bar: 50 m in A, 15 m in B and 65 m in D.

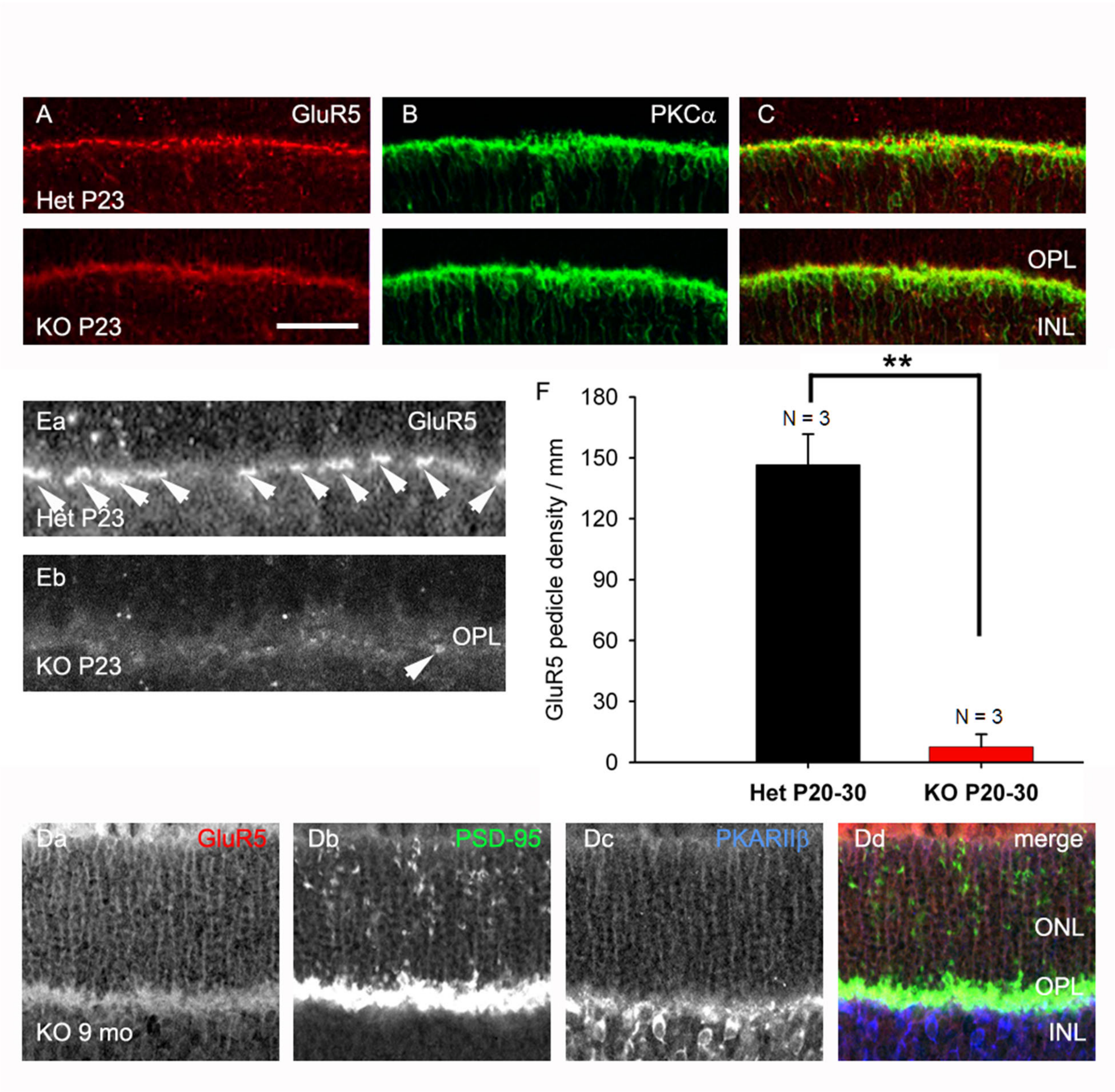

Figure S2:

Expression of ribbon presynaptic protein CtBP2 and rod bipolar cell marker PKCα is higher in VGLUT1 null retina (A) CtBP2 expression in VGLUT1 Het and null retina. CtBP2 expression is seen in both OPL and IPL, at the photoreceptor and bipolar cell presynaptic sites. The OPL and IPL of VGLUT1 null retina have higher density of CtBP2 puncta. A statistics could not be calculated for very high density of puncta. (B) Mean fluorescence of PKCα immunostained aged WT and VGLUT1 null INL is compared. VGLUT1 null retina has significantly higher PKCα indicating higher density of rod bipolar cells in the VGLUT1 null retina ($p < 0.01$). Scale bar: 50 μm.

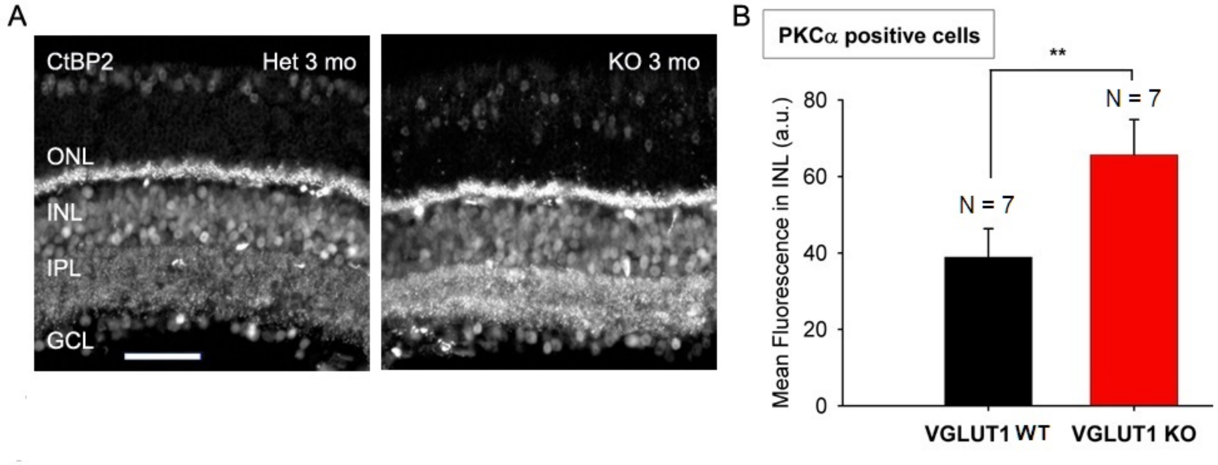

Figure S3:

Ganglion cell density has increased in the VGLUT1 null retina.

The mean fluorescent intensity of VGLUT2 immunoreactive ganglion cells in the GCL of retinal whole mount was measured using ImageJ and summarized for aged WT and VGLUT1 null (KO) groups. There is a little but significant increase in the mean fluorescent intensity in VGLUT1 null retina, mainly due to increased density of ganglion cells ($p < 0.01$).

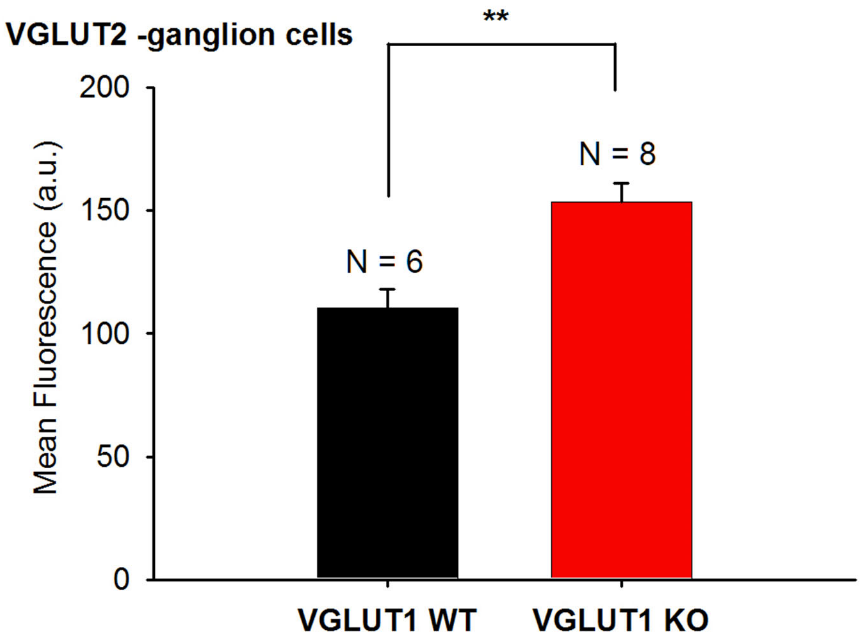

Figure S4:

Cholinergic wave in the juvenile VGLUT1 null retina. Cholinergic waves persist in the VGLUT1 null retina. Multielectrode array extracellular recording from a P25 VGLUT1 null retina is presented here as a rastar plot. Each row of the rastar corresponds to recording from a single unit, presumably a cell on the array. Recordings from 24 such cells are presented here. Vertical line in each row indicates an action potential. Propagating retinal waves of neurotransmitters cause propagation of depolarization, registered as action potential firing. Wave related action potential firings by cells are strongly correlated in time. Two of such temporally correlated action potential firings are marked with two leftmost red arrows. Application of ionotropic GABA and glycine receptor blockers gabazine (SR, GABA-A receptor blocker), TPMPA (GABA-C receptor blocker) and strychnine (Stry, glycine receptor blocker) causes disinhibition resulting in more frequent temporally correlated action potential firing. Red arrows indicate their occurrence. DHβE, a α4β2 nicotinic acetylcholine receptor blocker blocked these correlated action potential firing within 100 sec of application (right), proving these waves to be cholinergic.

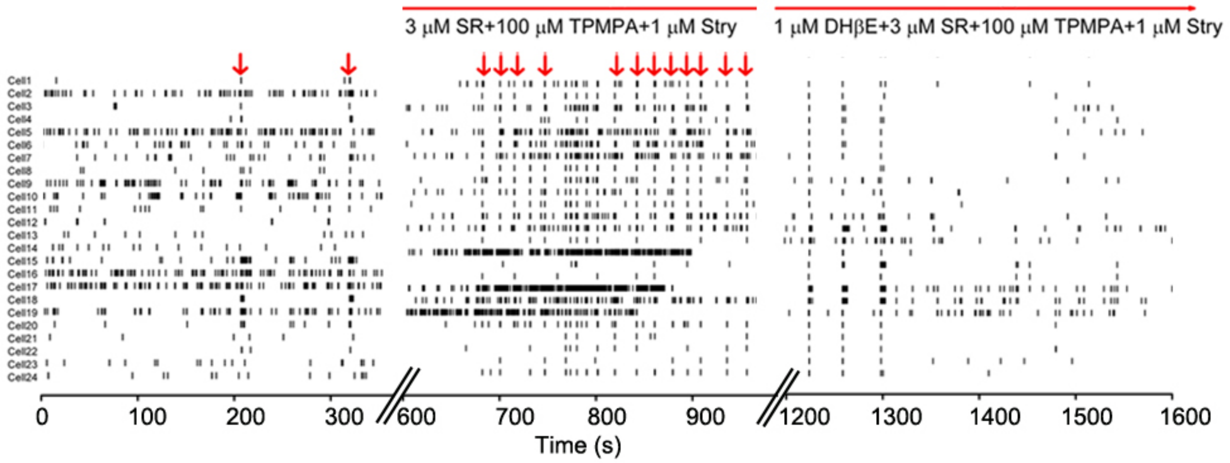

Figure S5:

Transient expression of VGLUT3 in cone pedicles of neonatal developing VGLUT1 Het retina

VGLUT3 expression is detected in the OPL of neonatal developing retina in both VGLUT1 Het and null littermates (Figure 11). We tried two different VGLUT3 primary antibodies, raised in rabbit and guinea pig, and 2 different secondary antibodies, Alexa 568 and Alexa 488, to confirm this transient expression. VGLUT2 was stained with Alexa 647 to make sure VGLUT3 and VGLUT2 channels were spectrally separated from each other and one did not bleed into another. (A) VGLUT3 stained with Alexa 568 and

VGLUT2 with Alexa 647 at P9 in the OPL of VGLUT1 WT retina. (B, C) VGLUT3 stained with Alexa 488 and VGLUT2 with Alexa 647 in P11 and P13 VGLUT1 WT retina. At P9-P11, VGLUT3 expression transiently increases in photoreceptors, and coincides with expression of VGLUT3 in them. By P13, strong VGLUT3 expression is gone from cone pedicles, but VGLUT2 continues to express, presumably since VGLUT1 co-expresses in these cones. (D) The same P13 retinas in C are visualized at the INL. VGLUT3 amacrine cells and some displaced VGLUT2 ganglion cells are seen. Scale bar: 50 μm.

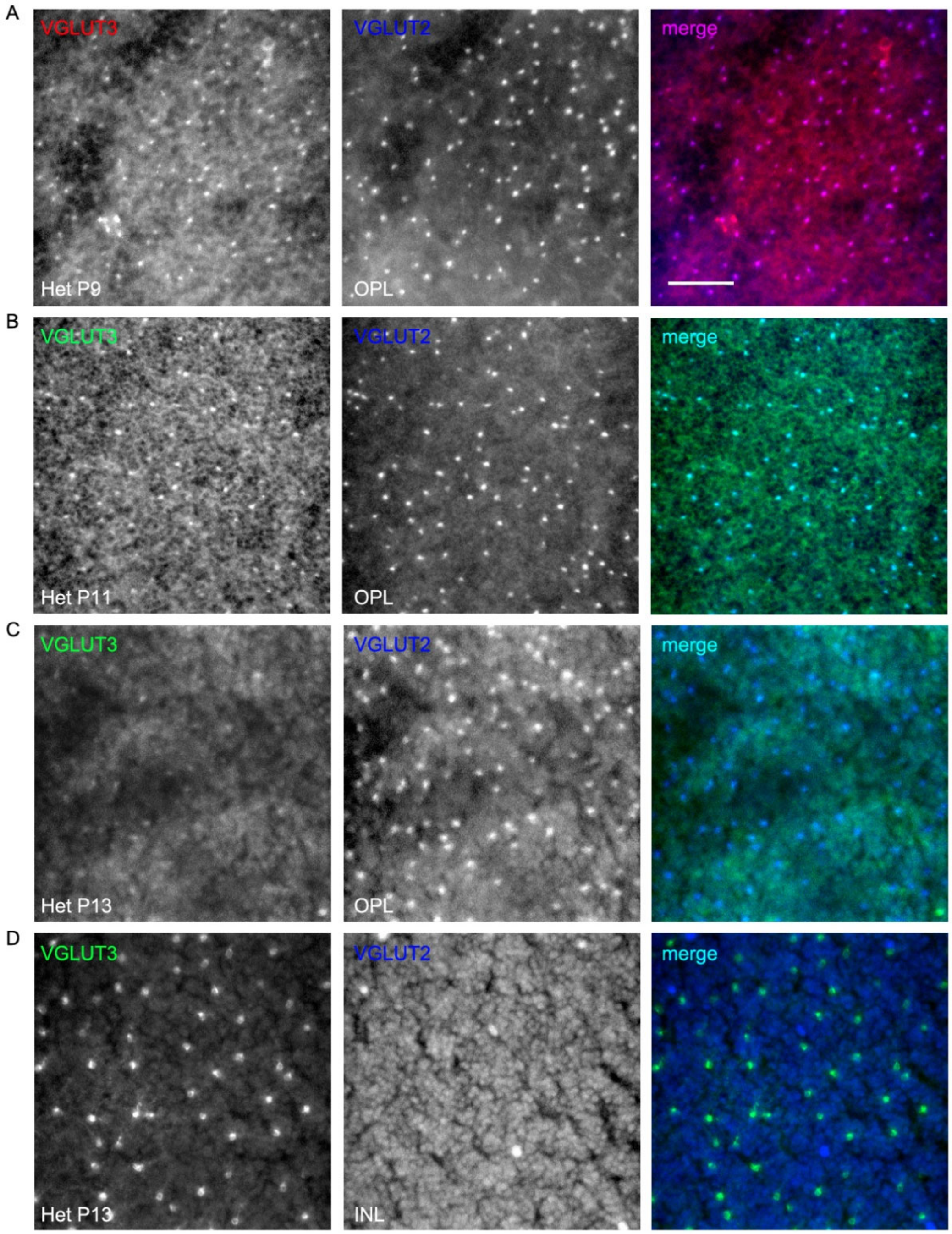

Figure S6:

Density of VGLUT2 cones is higher in juvenile/adult VGLUT3 null retina.

Three retinas each of VGLUT3 Het and null (KO) mice from the age group P13 – P45 were tested for expression of VGLUT2 in photoreceptors. The density of VGLUT2 cones appears to double in VGLUT3 null retina, compared to littermates ($p < 0.01$).

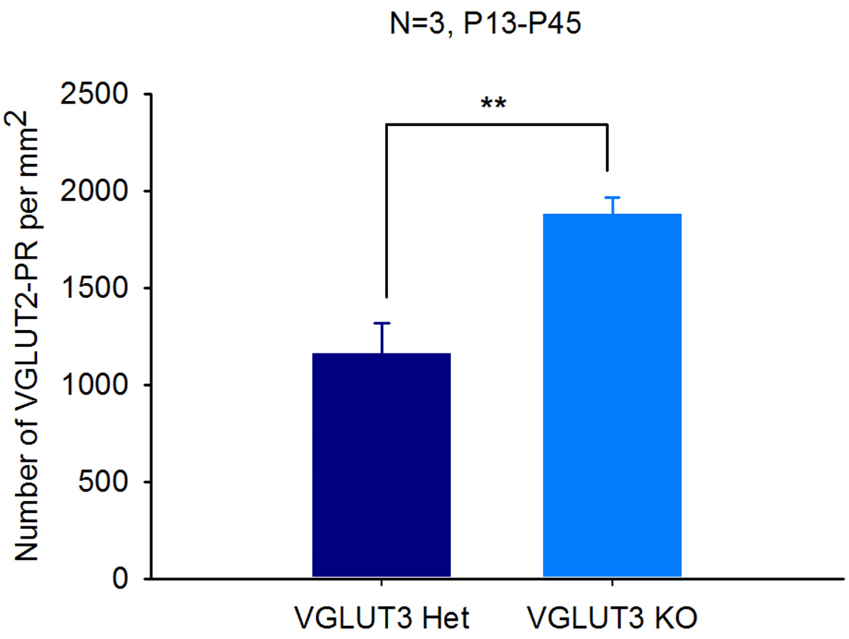
N=3, P13-P45
Number of VGLUT2-PR per mm^2
2500
2000
1500
1000
500
0
**
VGLUT3 Het
VGLUT3 KO
